# Development of a Vitamin K Database for Commercially Available Food in Australia

**DOI:** 10.3389/fnut.2021.753059

**Published:** 2021-12-09

**Authors:** Claire R. Palmer, Henrietta Koch, Sujata Shinde, Lauren C. Blekkenhorst, Joshua R. Lewis, Kevin D. Croft, Jonathan M. Hodgson, Marc Sim

**Affiliations:** ^1^Institute for Nutrition Research, School of Health and Medical Sciences, Edith Cowan University, Perth, WA, Australia; ^2^School of Biomedical Sciences, The University of Western Australia, Perth, WA, Australia; ^3^Medical School, Faculty of Health and Medical Sciences, The University of Western Australia, Perth, WA, Australia; ^4^Centre for Kidney Research, Children's Hospital at Westmead, School of Public Health, Sydney Medical School, The University of Sydney, Sydney, NSW, Australia

**Keywords:** food analysis, food composition, food database, Vitamin K1, Vitamin K2, phylloquinone, menaquinone

## Abstract

Vitamin K content of foods is known to vary substantially by geographical location. In Australia, no Vitamin K database of food exists, thereby creating ambiguity when trying to develop national dietary intake guidelines. This investigation aimed to develop a Vitamin K database for commonly consumed foods that are commercially available in Australian supermarkets. The Vitamin K1 (phylloquinone; PK) and K2 (menaquinone; MK4, MK7) content of 60 foods known to contain Vitamin K were assessed (e.g., vegetables fruits, oils, animal products, dairy and fermented foods). A liquid chromatography with tandem mass spectrometry (LCMS/MS) method was developed and used to measure PK and MKs in different foods with an improved chromatographic separation and detection of Vitamin K's and their analogs. The LOD and LOQ for PK and MK4 was 0.1, 0.5 ng/ml and 0.5, 1.0 ng/ml, respectively. The majority foods contained detectable PK (53/60), about half contained MK4 (31/60), and few contained MK7 (3/60). PK was highest in green leafy vegetables, with moderate amounts in oils. Highest MK4 content was in chicken eggs and meat products such as ham and chicken. This database enables nutritional epidemiologist to estimate dietary Vitamin K intake, especially in Australian cohorts, for a range of health outcomes.

## Introduction

Vitamin K refers to a group of fat-soluble vitamins best known for their role in blood coagulation. Other biological processes that Vitamin K has been implicated include blood calcium regulation, vascular anti-calcification, and bone metabolism (1). There are two main forms of Vitamin K; Vitamin K_1_ (phylloquinone; PK) and Vitamin K_2_ (menaquinones; MK). Phylloquinone is most abundant in green leafy vegetables and their oils, but is also present in smaller concentrations in the majority of food groups such as fruit, meat, and dairy products (2). In contrast to PK, the MKs are a group of isoprenologs where the side chain varies by the number of isoprenoid units ranging from four to thirteen repeats (MK4 to MK13; see Supplementary Figure 1) (3). Despite having analogous structures, the origins of menaquinones differ. MK4 is synthesized from PK by animals, and thus is obtained in the diet from animal products (3). All other MK are synthesized by anaerobic bacteria and thus are found in fermented foods, such as cheese (4).

Presently, there are several databases listing the PK content in a range of foods, especially vegetables and fruits. The most extensive of these databases has been developed by the United States Department of Agriculture (USDA) that has recently been updated (5). The USDA National Nutrient Database has recently been combined with the United Kingdom Composition of Foods Integrated Dataset (COFID) to provide an update to Vitamin K values in the Irish Food Composition Database (6). Although MK4 content of some food are listed, the USDA database does not include other MKs (MK5-12). Generally, such MKs are found in much lower quantities in meats and cheese compared to MK4, with the exception of MK7 known to be high in fermented foods such as Natto (7). Besides the USDA, MK reference ranges for food products are also limited to a few individual investigations (4, 7–10).

There are numerous methods to measure both PK and MK in biological matrices, as reviewed previously (11, 12). However, there is presently no standardized method to quantitate PK and MK in food. This has likely contributed toward a lack of detailed information on the Vitamin K content of food, which is reported to vary by regions including Europe, Asia and the United States of America by up to 50% (13). In Australia, a Vitamin K database for commonly consumed foods does not exist. This can limit nutritional epidemiology when researchers seek to investigate the potential health benefits of Vitamin K (PK and/or MK) for a range of health outcomes in Australian cohorts. Since MKs are estimated to constitute about 10% of total dietary Vitamin K intake, with the majority (up to 40%) of dietary MK attributed to MK4 (3, 14), the aim of this investigation was to develop a preliminary Vitamin K database, assessing PK and MK4 for commonly consumed commercially available foods in Australia.

## Methods

### Database Creation

Food items were primarily selected based on the foods assessed in a commonly used Australian food frequency questionnaire (FFQ) developed by the Cancer Council of Victoria (DQES V2) (15, 16). This FFQ is designed to cover dietary intake over a period of 12 months, a timeframe thought to represent the “usual” diet. From the 101 foods and beverages (including alcohol) recorded in the FFQ, this was subsequently condensed to 60 food items which were known to contain PK and MK4 or MK7, based on previous work (8–10, 17). The main categories of food groups considered included vegetables (*n* = 20), fruits (*n* = 3), oils (*n* = 4), animal products (*n* = 16), dairy (*n* = 14) and fermented foods (*n* = 3). This list also included food items unique to Australia [kangaroo, yeast extract spread (Vegemite)]. It was beyond the scope of the current investigation to explore all other MKs in the selected foods. As previously highlighted, MKs are estimated to constitute about 10% of total dietary Vitamin K intake, with the majority (up to 40%) of dietary MK attributed to MK4 (3, 14). Hence, we were primarily concerned with assessing MK4 content in food. Nevertheless, we also quantified MK7 which is known to be present in food such as fermented vegetables and dairy.

Three leading local supermarket franchises were visited to obtain one sample (or brand) of food item from each store. This resulted in up to three samples per food item obtained for analysis, depending on availability. Vegetable, fruit, and meat products were processed in a food processor (Multichopper, Sunbeam Australia) prior to storage. The food samples were stored in 5 ml freezer tubes at −80°C until extraction. Every food item obtained was subsequently analyzed in duplicate. For example, if three separate brands of full fat milk were obtained, a total of six samples would have been analyzed. Once analyzed, the median value was calculated to provide an estimate of the content of Vitamin K in the analyzed food item.

#### Chemicals and Reagents

Chemicals used for extraction were of HPLC grade and solvents used for chromatography were of LCMS grade. Chemicals: dichloromethane (VWR International Ltd, Tingalpa, QLD), absolute ethanol (VWR International Ltd, Tingalpa, QLD), ammonium formate (Sigma Aldrich, Castle Hill, NSW), formic acid (Univar, Sydney, NSW) n-hexane (Fisher Scientific, Loughborough, England), methanol (Fisher Scientific, Loughborough, England), isopropanol (Fisher Scientific, Loughborough, England) and diethyl ether (Sigma Aldrich, Castle Hill, NSW) were all used as received.

Deuterium-labeled Vitamin K standards: PK-d_7_ (5,6,7,8-d_4_,2-methyl-d_3_), MK4-d_7_ (MK4)-(5,6,7,8-d_4_,2-methyl-d_3_) and MK7-d_7_ (MK7)-(5,6,7,8-d_4_,2-methyl-d_3_) were purchased from Sigma Aldrich (St Louis, USA), each with chemical purity of ≥95%, and ≥98 atom % deuterium. Non-deuterated PK and MK4 were purchased from Sigma Aldrich (St Louis, USA). Non-deuterated MK7 was unavailable to our lab.

#### Vitamin K Extraction

The method of Vitamin K extraction was adapted from prior published methods (18–20). All procedures were performed under yellow light to reduce the photo-oxidation of Vitamin K by UV light. Approximately 0.2 g of the food was weighed into 15 ml screw-capped polyethylene centrifuge tubes and spiked with PK-d_7_ (10 ng), MK4-d_7_ (10 ng) and MK7-d_7_ (15 ng). Proteins were denatured with ethanol (1 ml), followed by a 1 min wait period. Extraction was performed with hexane (2 ml) and Millipore H_2_O (1 ml). The samples were vortexed (1 min) and shaken (3 min), followed by further agitation in an ultrasound bath (10 min), and gyratory mixer (20 min). Meat samples were also sonicated for 30 s, to further homogenize the sample (Branson Ultrasonics Corporation, Sonicator 150). Samples were centrifuged at 1,800×g for 10 min at 25°C. The upper organic layer was removed and purified on 3 ml silica columns (Agilent Technologies Bond Elute, Mulgrave, VIC) according to Tarvainen et al. (21). Since Vitamin K is fat soluble, foods with a high fat content used a high capacity column (Agilent Technologies, Bond Elute silica, 10 g, 60 ml, 120 μm, Mulgrave, VIC), subsequently, extraction volumes were doubled, and the columns were preconditioned with 60 ml diethyl ether, washed with 60 ml hexane, and eluted with 54 ml of hexane: diethyl ether (3.5: 96.5%). The purified solution was collected into glass tubes then dried under nitrogen at 50°C. The residue was dissolved in dichloromethane (20 μl), dried under nitrogen, and then heated for 10 mins at 60°C to remove any residual solvent. Samples were then reconstituted in isopropanol (200 μl) and placed in amber vials for analysis by LCMS/MS.

#### Liquid Chromatography With Tandem Mass Spectrometry (LCMS/MS) Parameters

The extracted phylloquinone and menaquinones were analyzed on a Thermo Scientific TSQ Quantum Ultra Triple Quadrupole mass spectrometer equipped with a heated ESI source. It was operated in the positive ion mode and connected to an Accela Autosampler system. Chromatographic separation was performed on a reverse-phase Accucore PFP HPLC column (2.6 μm, 100 × 2.1 mm). The mobile phase consisted of methanol containing 0.1% formic acid (solvent A) and 5 mM ammonium formate with 0.1% formic acid (solvent B). Column and tray temperature was maintained at 40 and 35°C, respectively. The run time for the LC method was 6 mins and the solvent gradient conditions were as follows, 90% A at 0 time, isocratic at 90% A for 1 mins, 100% A at 1.01 mins and held at 100% A to 5 mins, then reduced to 90% A at 5.01 mins and held at 90% A for 6 mins to equilibrate to starting conditions. The flow rate was 0.5 ml/min, with a 10 μl injection volume. The mass spectrometer was used in the multiple reaction monitoring mode with argon as the collision gas to detect PK, PK-d_7_, MK4, MK4-d_7_, MK7 and MK7-d_7_, with MS parameters listed in Table 1. For quantitative analysis, ratios of area of peak for PK and MK4, to their deuterated standards at the respective RTs, to known amount of added internal standards, PK-d_7_ and MK4-d_7_ were used to calculate concentration in μg and expressed as per 100 g of food matrix. The concentration of MK7 in food samples was determined by measuring the area under the peak of non-deuterated precursor ion, transition *m/z* 650 > product ion *m/z* 187 based on the RT of the deuterated internal standard, MK7-d_7_, transition *m/z* 657.1 > *m/z* 194.2 (Supplementary Figure 2). The ratio of area of peaks for MK7 and MK7-d_7_, assuming a 1:1 response, to a known amount of MK7-d_7_ (15 ng) added during the analysis was used to calculate the concentration of MK7 in μg and expressed as per 100 g of food matrix.

**Table 1:** MRM transitions and RT for deuterated and non-deuterated phylloquinone (PK), menaquinone-4 (MK4), and menaquinone-7 (MK7).

	**Precursor ion**	**Product ion**	**Collision Energy**	**RT** **mins**
	***m/z***	***m/z***	**eV**	
PK	451.0	187.0	25	1.90
PK-d_7_	458.4	194.2	23	1.91
MK4	445.0	187.0	20	1.53
MK4-d_7_	452.0	194.0	25	1.57
MK7	650.0	187.0	26	-
MK7-d_7_	657.1	194.2	28	2.30

*RT, retention time*.

#### Method Validation

Linearity, limit of detection (LOD), and limit of quantification (LOQ) were determined for the vitamin PK and MK4 by using incrementally diluted calibration standards ranging from 0.01 to 500 ng/ml. Linear regression analysis was conducted using the ratio of the K vitamin to deuterated internal standard (PK-d7 or MK4-d7) as a function of concentration of the K vitamin. The R^2^ for both PK and MK4 was 0.9987 (Supplementary Figure 3). LOD and LOQ was determined in accordance with the US Food and Drug Administration guidelines (22). The LOD and LOQ for PK was 0.1 and 0.5 ng/ml. respectively. The LOD and LOQ of MK4 was 0.5 and 1 ng/ml, respectively. Precision was determined by calculating the % CV of PK and MK4 concentrations in standard solutions and was repeated over three consecutive days to determine intra- and inter-assay variability. Intra and inter-assay variability for phylloquinone was 10.6 and 12.8%, respectively. Intra and inter-assay variability for MK4 was 3.9 and 10.1%, respectively.

Due to the wide range of food matrices investigated precision was not determined in all matrices. As such, a simulated matrix was used to determine recovery. Recovery was determined for PK and MK4 by spiking a low-fat milk matrix with known amounts of PK, PK-d7, MK4, and MK4-d7. In two tubes a known amount of the standards was added at the start of the extraction process and in three separate tubes the same amount of standard was added at the end of the extraction process, with the recovery determined by comparing the measured concentrations of PK and MK4 in samples spiked before extraction to those spiked following purification. Recovery for PK was 41.3% and for MK4 was 43.4%; the recovery for PK-d7 and MK4-d7 was 37.0 and 40.2%, respectfully, resulting in approximately equal ratios. MK7 was not included in these experiments due to lack of a MK7 standard.

## Results

### Food Database

The PK, MK4, and MK7 content for the individual foods analyzed are displayed in Table 2. All PK and MK4 measurements were higher than the LOQ. The selective reaction monitoring chromatograms of PK, MK4 and MK-7 in selected foods (including spinach, cheese and natto, respectively) are presented in Supplementary Figure 2. The majority of assessed foods contained detectable PK (53/60), just over half contained MK4 (31/60), and few contained MK7 (3/60).

**Table 2:** Median (range) values of the phylloquinone and menaquinone content of commonly consumed foods obtained from Australian supermarkets.

**Food item**	**Phylloquinone** **(μg/100 g)**	**MK4** **(μg/100 g)**	**MK7** **(μg/100 g)**
			
**Vegetables**			
Spinach	262.9 (244.7–286.2)	ND	ND
Kale	128.5 (92.5–272.6)	ND	ND
Cabbage	70.4 (28.6–94.4)	ND	ND
Broccoli	67.9 (50.9–92.9)	ND	ND
Carrot	36.4 (10.0–67.5)	ND	ND
Green beans	32.5 (29.1–46.7)	ND	ND
Cucumber	26.4 (17.1–32.0)	ND	ND
Lettuce	25.7 (10.3–43.2)	ND	ND
Peas	22.7 (17.9–31.4)	ND	ND
Kidney beans	21.0 (5.4–29.7)	ND	ND
Zucchini	18.3 (12.8–24.7)	ND	ND
Celery	17.3 (13.6–20.8)	ND	ND
Cauliflower^#^	16.4 (12.5–20.1)	ND	ND
Pumpkin	14.8 (3.3–19.3)	ND	ND
Capsicum	9.55 (8.2–14.1)	ND	ND
Tofu^#^	7.0 (5.9–8.1)	ND	ND
Tomato	6.0 (4.4–7.3)	ND	ND
Bean shoots^*^	5.4 (5.1–5.7)	ND	ND
Potato	0.5 (0.2–0.7)	ND	ND
**Fruits and nuts**			
Avocado	23.6 (16.2–31.4)	ND	ND
Cashews	12.3 (5.4–14.0)	ND	ND
Pear	1.8 (1.4–2.7)	ND	ND
Apple	1.4 (1.2–2.2)	ND	ND
**Oils**			
Canola oil	75.0 (64.6–121.4)	ND	ND
Margarine	69.2 (53.4–80.1)	6.41 (3.9–8.1)	3.5 (0.0–37.8)
Olive oil	43.6 (38.6–45.1)	ND	ND
Butter	5.10 (3.5–5.6)	24.69 (30.9–30.4)	ND
**Animal products**			
Egg	3.34 (1.9–4.4)	32.61 (22.5–37.9)	ND
Ham	ND	28.78 (22.1–45.7)	ND
Chicken	0.3 (0.2–0.6)	26.4 (18.5–31.8)	ND
Salami	0.3 (0.0–0.9)	18.18 (16.0–42.0)	ND
Pork	ND	15.9 (8.5–19.1)	ND
Beef	0.6 (0.4–1.6)	15.6 (5.9–21.6)	ND
Beef mince	1.2 (0.5–1.8)	13.7 (12.2–17.8)	ND
Bacon	ND	12.72 (9.5–13.0)	ND
Lamb	0.4 (0.3–0.5)	11.6 (7.8–14.9)	ND
Sausage (beef)	0.3 (0.2–0.7)	3.0 (2.1–9.7)	ND
Barramundi	0.2 (0.0–0.4)	2.16 (1.5–2.7)	ND
Tinned salmon	0.4 (0.3–0.7)	2.0 (1.4–4.4)	ND
Tinned tuna	ND	1.4 (1.1–2.0)	ND
Kangaroo^*^	0.17 (0.13–0.21)	1.1 (1.0–1.2)	ND
Veal^*^	0.1 (0.1–0.2)	1.0 (0.9–1.1)	ND
Snapper^#^	ND	0.8 (0.7–1.1)	ND
**Dairy**			
Thickened cream	ND	20.1 (18.2–29.8)	0.9 (0.4–3.1)
Sour cream	3.0 (2.6–4.4)	12.72 (10.9–16.4)	ND
Brie	1.84 (1.1–2.9)	9.34 (3.7–11.3)	ND
Cheddar	1.1 (0.8–1.2)	5.5 (4.6–5.9)	ND
Parmesan	0.7 (0.4–1.0)	5.4 (3.3–8.0)	ND
Cream cheese	1.3 (0.7–1.9)	5.0 (3.4–10.8)	ND
Low fat cheddar	0.7 (0.5–0.9)	4.1 (3.4–4.7)	ND
Ice cream	2.3 (0.0–5.3)	4.07 (1.8–6.7)	ND
Greek yogurt	0.6 (0.0–1.2)	3.5 (0.0–5.1)	ND
Full fat milk	1.3 (1.1–2.7)	1.21 (1.0–1.8)	ND
Yogurt	0.3 (0.2–0.5)	1.0 (0.5–3.0)	ND
Cottage cheese	0.4 (0.3–0.5)	0.97 (0.6–1.1)	ND
Reduced fat milk	0.21 (0.0–0.3)	ND	ND
Skim milk	ND	ND	ND
**Fermented foods**			
Sauerkraut^*^	7.0 (3.2–10.7)	ND	ND
Natto^*^	6.2 (6.0–6.4)	ND	81.6 (68.8–94.5)
Yeast extract^*^ spread (vegemite)	2.34 (1.9–2.8)	(1.4–8.6)	ND

* Indicates mean value; ND, not detected. Three separate samples were analyzed for all food items apart from those indicated by # or *, where two or a single food item was analyzed, respectively. Each sample of food was also assessed in duplicate. Where only one food sample was analyzed (in duplicate), the mean and range has been presented instead.

**Figure 1:**
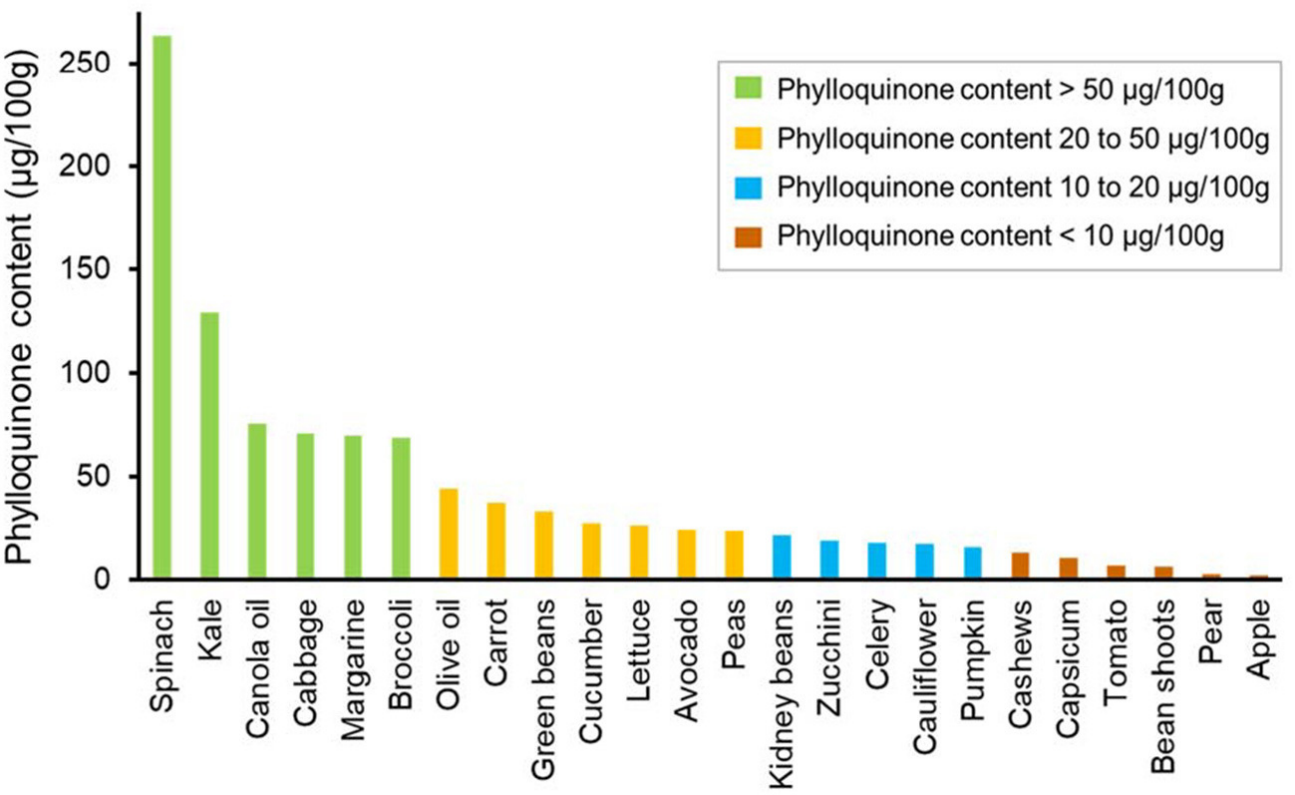
Median phylloquinone content of individual vegetables, vegetable oils, and fruits from highest to lowest.

Phylloquinone was detected in all vegetables, being highest in green leafy vegetables such as spinach (median 263.0 μg/100 g) and kale (median 128.5 μg/100 g) (Figure 1). Phylloquinone was of lower abundance (<25 μg/100 g) in fruit. MK4 and MK7 were not detected in any non-fermented vegetable or fruit. Japanese fermented soybeans (Natto), contained the highest content of MK7 (mean 81.6 μg/100 g).

Vegetable oils contained moderate amounts of PK. Noteworthy, margarine contained moderate amounts of PK, MK4 and in some samples MK7. Butter contained smaller amounts of PK (5.1 μg/100 g), but a high abundance of MK4 (24.7 μg/100 g).

**Figure 2:**
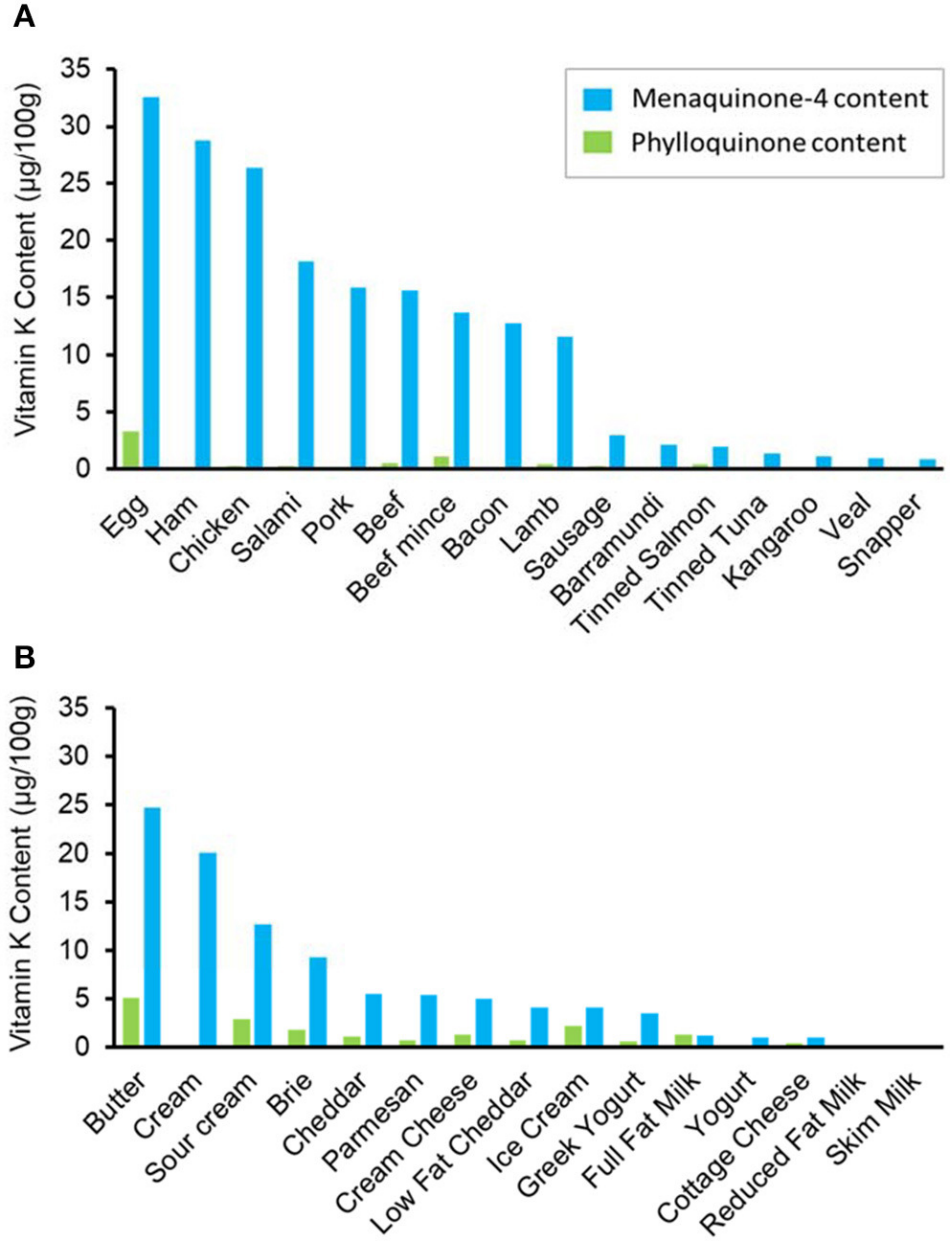
Median menaquinone-4 (blue) and phylloquinone (green) content of **(A)** meat products and **(B)** dairy products. Presented from highest to lowest menaquinone content.

The majority of animal products, with exception of reduced fat and skim milk, contained MK4 (Figure 2). The highest MK4 content was in chicken eggs (32.61 μg/100 g) and meat products such as ham (28.8 μg/100 g) and chicken (26.4 μg/100 g). MK4 content was low in fish products (<5.0 μg/100 g) and kangaroo meat (1.1 μg/100 g). The PK content of all animal products was minimal (<6.0 μg/100 g). Finally, only a small amount of MK7 was detected in thickened cream (0.9 μg/100 g).

## Discussion

For the first time, we present an Australian food composition database for Vitamin K. Specifically, we present the PK, MK4 and MK7 content of 60 commonly consumed commercially available foods. Food items analyzed comprised a wide range of food groups known to provide the majority of dietary Vitamin K1 and K2, including vegetables, oils, meat, and dairy. We were able to accomplish this by adopting an LCMS/MS method to assess the Vitamin K content of these food items. Findings of this work may help with the revaluation of Vitamin K intake guidelines in Australia where an adequate intake of 60 and 70 μg/day for men and women, respectively, is proposed (23), which are substantially lower than the USA (120 μg/day for men, 90 μg/day for women) (24).

Similar to previous work, vegetables were found to be a major source of PK. We report that specific vegetables including spinach and kale as the richest sources of PK (263, 129 μg/100 g, respectively). Noteworthy, the PK content of these vegetables differed substantially compared to previous work. For example, Schurgers and Vermeer report that PK in spinach and kale to be 387 and 817 μg/100 g. Alternatively, the USDA report these measurements to be 483 and 390 μg/100 g, respectively. Compared to these studies, PK content in spinach we measured varied between 33 and 45%. For kale, the PK content was approximately 6 times lower compared to Schurgers and Vermeer (8), and 3 times lower compared to the USDA. Nevertheless, it has been suggested that the average PK content in “green vegetables” to be in the range of 100–750 μg/100 g (14). Although canola oil and margarine were shown to contain a high-moderate amount of PK, these foods are typically consumed in quantities less than 100 g/day. Therefore, such foods may not be a large contributor toward overall dietary Vitamin K intake. Collectively, these results highlight that the importance of accounting for regional differences in PK content of food to reduce inaccuracies when trying to estimate PK intake for specific populations. Noteworthy, the Vitamin K content of fruits are reported to be low (25, 26), a finding we also observed in apples and pears in this study. Although it would have been ideal to assess the Vitamin K content of more fruits, due to limited resources, we selected some of the most commonly consumed fruits previously shown to contain Vitamin K.

Others including ourselves have now demonstrated that the MK4 in foods also differ between regions (13). Specifically, MK4 content of beef cuts from the USA (1.1–9.3 μg/100 g) was substantially lower compared to Japan (15.0 ± 7.0 μg/100 g) (9, 18). Egg yolk MK4 content was also 4 times greater in Japan compared to the USA (64 vs. 15.5 μg/100 g, respectively). In comparison, our measurements in whole egg and beef indicated a MK4 content of 33 and 16 μg/100 g, respectively. Such regional differences may be attributed to differences in food production including the use of menadione in animal feeds (13). Hard and soft cheese are also known to be dietary sources of MKs (4, 13) that provide between 4 and 10 μg/100 g (13). These findings are consistent with our results where MK4 content of brie, cheddar and parmesan were 9, 6 and 5 μg/100 g, respectively. Although not assessed, it is worth highlighting that up to 15% of variability has been reported in the MK content (MK-6 to MK-10) of semi-hard cheese varieties from three different European countries, including France, Poland and Denmark (4). Similar to previous work, MK7 was not detected (or found in very small amounts) in cheese varieties (13), whilst margarine was found to contain almost double the amount of MK7 compared to MK4 (6 vs. 11 μg/100 g, respectively). In line with previous work, natto was found to be a rich source of MK7 (82 μg/100 g). However, this was substantially lower compared to the MK7 content previously measured (902–998 μg/100 g) (9, 13, 21).

In order to create food databases, a validated method of measuring PK and MKs simultaneously, with accuracy and high throughput, is required. This is further complicated as the Vitamin K content in foods are typically low, in conjunction with the added complexity of extraction from the food matrix (27). We have previously provided an overview of the current methods for analyzing Vitamin K compounds in food (12). An emerging method to measure Vitamin K in foods is a HPLC-MS method developed by Karl et al. (20), which has high versatility and may be used to measure PK and all MKs in a variety of forms such as food, serum and feces. A combination of HPLC and gas chromatography-MS (GC-MS) has been used with deuterium-labeled internal standards to accurately measure PK in serum (28). However, there are limitations in using GC for measuring Vitamin K, as high temperatures are needed to volatise Vitamin K compounds (>300°C) (29). Therefore, HPLC is the preferred method to separate Vitamin K compounds for analysis. Previously, reverse-phase HPLC with fluorescent detection or HPLC–MS, with K1 or deuterated-K1 as the internal standard have been used to determine PK and MK from food (4, 20). Most recently, a whole range of fermented foods were assessed for their PK and MK content using a newly developed ultra-high performance liquid chromatography–atmospheric pressure chemical ionization tandem mass spectrometric (UHPLC-APCI-MS/MS) method (21). They reported a LOD and LOQ for PK and MK4 of 1.0, 3.2 pg and 6.4, 21.2 pg, respectively. In the present study, we used HPLC method coupled to heated electrospray ionization tandem mass spectrometry (H-ESI-MS/MS) to measure PK and MK in different foods with an improved chromatographic separation and detection of Vitamin K's and their analogs with the LOD and LOQ for PK and MK4 as 1 and 5 pg (0.1 and 0.5 ng/ml) and 5 and 10 pg (0.5 and 1 ng/ml), respectively. These values are comparable to that reported by Tarvainen et al. (21). In comparison, we have developed an efficient LCMS/MS method with a shorter chromatography run time for the quantification of PK and MK4 with the use of deuterated internal standards. Specifically, when adopting a modified LCMS/MS method here (30), we recorded faster elution of PK and MK4 retention times (1.90 and 1.53 mins), compared to previous work analyzing fermented foods (5.52 and 4.66 mins) (21) as well as human serum and plasma (12.20 and 10.41 mins) (19).

In conclusion, we have provided the Vitamin K (PK, MK4 and MK7) content of commonly consumed commercially available food products in Australia. The LCMS/MS method enabled the quantification of PK, MK4, and MK7 in these food products with acceptable LOD and LOQ and inter and intra-assay precision. In conjunction with other Vitamin K food database, this data can be used by nutritional epidemiologist seeking to quantify dietary intake, especially in Australian cohorts, for a range of health outcomes.

## Data Availability Statement

Within reasonable request, the raw data supporting the conclusions of this article will be made available by the authors.

## Author Contributions

MS, LB, JL, KC, and JH conceived and designed the study. CP and HK performed the analysis. SS and KC did the LCMS/MS method development. CP and MS prepared the manuscript. CP had the primary responsibility for the final content. All authors reviewed, read, and approved the final manuscript.

## Funding

This research was supported by a Royal Perth Hospital Research Foundation Springboard Grant. The salary of MS is supported by a Royal Perth Hospital Research Foundation Career Advancement Fellowship (CAF00/2020). The salary of LB is supported by a National Health and Medical Research Council (NHMRC) of Australia Emerging Leadership Investigator Grant (ID: 1172987) and a National Heart Foundation of Australia Post-Doctoral Research Fellowship (ID: 102498). The salary of JL is supported by a National Heart Foundation of Australia Future Leader Fellowship (ID: 102817). The salary of JH is supported by a National Health and Medical Research Council of Australia Senior Research Fellowship (ID: 1116973). None of the funding agencies had any role in the conduct of the study; collection, management, analysis, or interpretation of the data; or preparation, review, or approval the manuscript.

## Conflict of Interest

The authors declare that the research was conducted in the absence of any commercial or financial relationships that could be construed as a potential conflict of interest.

## Publisher's Note

All claims expressed in this article are solely those of the authors and do not necessarily represent those of their affiliated organizations, or those of the publisher, the editors and the reviewers. Any product that may be evaluated in this article, or claim that may be made by its manufacturer, is not guaranteed or endorsed by the publisher.
