# Supplementary material for: Development of a Vitamin K Database for Commercially Available Food in Australia
Source: Front Nutr. 2021 Dec 9;8:753059. doi: 10.3389/fnut.2021.753059 (PMC8698136; doi:10.3389/fnut.2021.753059)
Supplement: Supplementary file 1 [file Data_Sheet_1.docx]

**
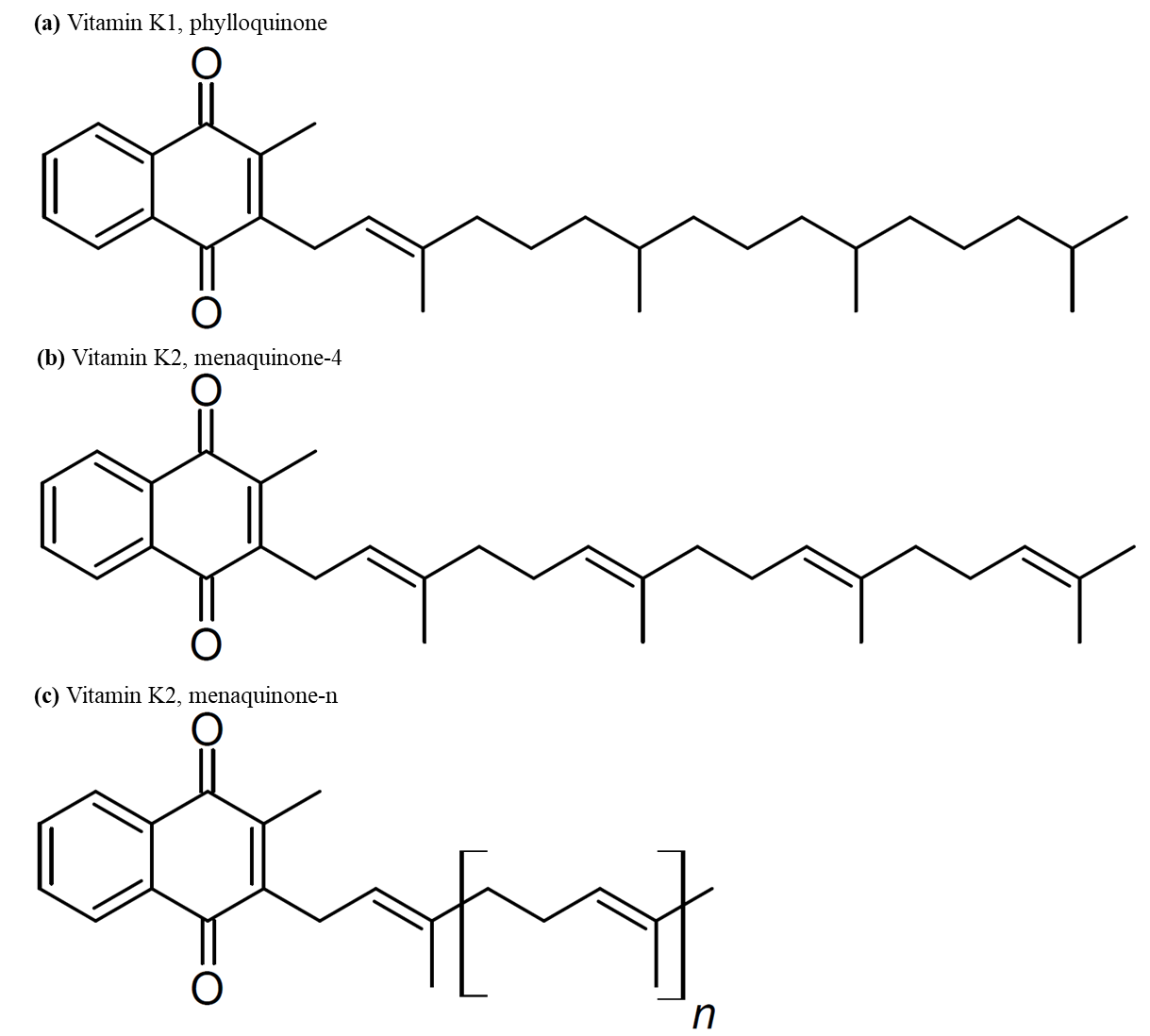
**

**Supplementary Figure 1**. Chemical structure of vitamin K 1 and K2 compounds, including

the menaquinone derivatives. (adapted from Palmer et al. 2020 with permission).


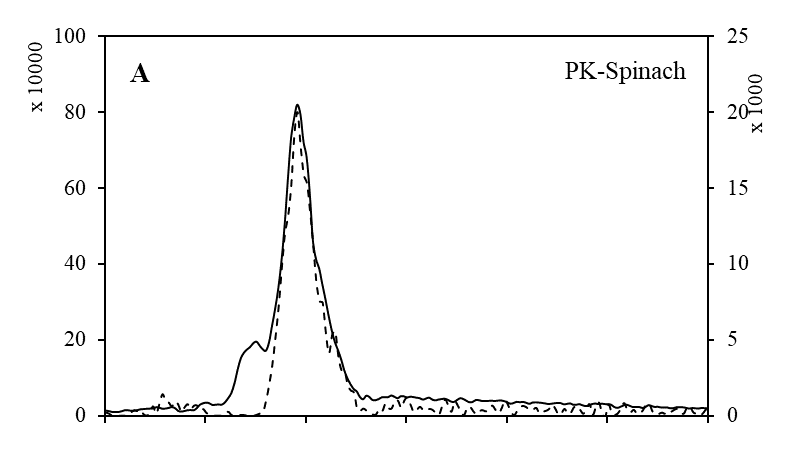

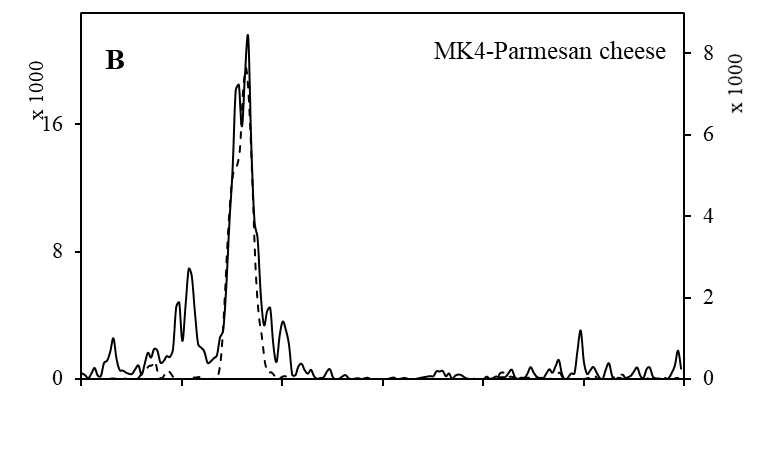

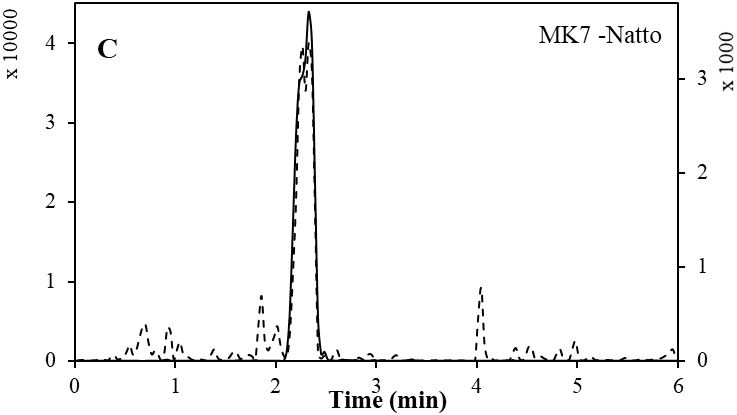


**Supplementary Figure 2.** Representative SRM chromatograms in positive ion mode of Vitamin K and its analogues in some Australian foods (**A**) Spinach, Phylloquinone (**PK**) (solid line), *m/z* transition 451 > *m/z* 187.0 and PK-d_7_ (dashed lines), *m/z* transition 458.4 > *m/z* 194.2 (**B**) Cheese (Parmesan), Menaquinone, (**MK4**) (solid line), *m/z* transition 445.0 > *m/z* 187.0 and MK4-d_7_ (dashed lines), *m/z* transition 452.0 > *m/z* 194.0 and (**C**) Natto, **MK7** (solid line), *m/z* transition 650 > *m/z* 187 and MK7-d_7_ (dashed lines), *m/z* transition 657.1 > *m/z* 194.2.

**Supplementary Figure 3.** Linear regression plots for (**A**) Phylloquinone, PK and (**B**) Menaquinone-4, MK4.
